# The bone-cerebrovascular axis: effects of bone aging on neurovascular dysfunction and neurodegeneration

**DOI:** 10.1172/JCI207273

**Published:** 2026-07-15

**Authors:** Jiekang Wang, Xu Cao, Mei Wan

**Affiliations:** 1 Department of Orthopaedic Surgery and; 2 Department of Biomedical Engineering, The Johns Hopkins University School of Medicine, Baltimore, Maryland, USA.

## Abstract

Beyond serving as a structural organ, the skeleton undergoes continuous remodeling and functions as an endocrine organ by secreting bioactive factors that regulate the physiology of distant tissues. Indeed, the concept of a “bone-vascular axis” has long been recognized, supported by epidemiological evidence linking osteoporosis and low bone mass to increased cardiovascular morbidity and mortality. Emerging findings now extend this paradigm to the brain, suggesting that bone- and bone marrow–derived signals influence cerebrovascular structure, function, and aging. Given that cerebrovascular dysfunction is a central driver of age-related cognitive decline, dementia, and neurodegenerative diseases, understanding this “bone-cerebrovascular axis” may offer novel opportunities for prevention and intervention. Here, we outline the cellular and molecular mechanisms underlying age-associated neurovascular impairment and summarize the biology of major bone and bone marrow cell populations, with emphasis on age-related alterations in their secretome. A central focus of this Review is the emerging evidence that age-related skeletal alterations exert systemic effects on the cerebrovasculature, highlighting how bone- and bone marrow–derived factors shape neurovascular health and pathology, which may subsequently contribute to CNS aging and neurodegeneration. A deeper understanding of these systemic interactions reframes brain aging within a whole-body context and may uncover innovative biomarkers and therapeutic strategies to mitigate neurodegeneration and other age-associated disorders.

## Introduction

The past decade has witnessed a rapid explosion in our understanding of age-related neurodegenerative disorders, including Alzheimer’s disease (AD), vascular dementia, frontotemporal dementia, and Parkinson’s disease, providing important insights into cell-intrinsic and organ-specific mechanisms. More recently, attention has shifted toward systemic regulation, with growing evidence indicating that age-associated circulating factors derived from peripheral organs act as key drivers of neurological decline and cognitive impairment. Heterochronic parabiosis and heterochronic plasma transfer experiments have identified multiple circulating pro-geronic factors as contributors to brain aging (1–8), suggesting that distal organs regulate brain aging through the secretion of circulating factors.

Among the most important sources of these factors is the skeleton, which is a highly dynamic organ that undergoes continuous remodeling throughout life. Although traditionally recognized for its functions in structural support, protection, movement, mineral homeostasis, and hematopoiesis, the skeleton is now increasingly appreciated as an active endocrine organ (9–12). Bone- and bone marrow–derived cells, including osteoblasts, osteocytes, osteoclasts, and bone marrow adipocytes, secrete hormones and cytokines that influence systemic processes, such as energy metabolism, mineral homeostasis, vascular function, and brain activity (5–7, 9, 12, 13). During aging and in pathological conditions, the skeletal secretome undergoes profound alterations, characterized by elevated levels of pro-inflammatory cytokines and the emergence of a bone cell–associated senescence-associated secretory phenotype (bone-SASP) (14, 15). These changes not only contribute to skeletal degeneration but also may promote dysfunction in distal organs.

The “bone-vascular axis” has long been recognized, with substantial epidemiological, clinical, and experimental evidence supporting a close association between bone metabolism and vascular health (16–19). However, most of those studies focused on extracranial large vessels. More recent studies have begun to examine the relationship between skeletal aging and the cerebrovasculature, which is the primary focus of this Review. In parallel, emerging evidence also supports a conceptual framework of a “bone-brain axis.” Bone communicates with the brain through two complementary pathways. First, the brain can perceive and respond to signals from bone tissue through a process termed “skeletal interoception,” where ascending signals are sent from bone tissue via sensory nerves to the CNS, and the descending signals from the CNS regulate bone and adipose metabolism via the sympathetic nervous system (12, 20–36). Second, bone and bone marrow cell–secreted factors are released into the bloodstream to regulate brain functions.

Here, we do not aim to comprehensively cover bidirectional bone-brain interactions or the broader bone-vascular interaction; rather, we focus on how bone, as an endocrine organ, regulates cerebrovascular function and aging. We first outline the mechanisms underlying cerebrovascular impairment in aging and age-associated neurodegenerative diseases, with an emphasis on blood-brain barrier (BBB) dysfunction. We summarize clinical evidence for bone-cerebrovascular interactions and discuss how bone-targeting interventions may influence cerebrovascular disorders. We further highlight how age-related skeletal changes may impair cerebrovascular integrity through circulating pro-geronic factors, offering potential biomarkers and therapeutic targets to prevent neurovascular dysfunction and neurodegeneration.

## Cerebrovascular homeostasis and dysfunction in aging and neurodegeneration

**Figure 1:**
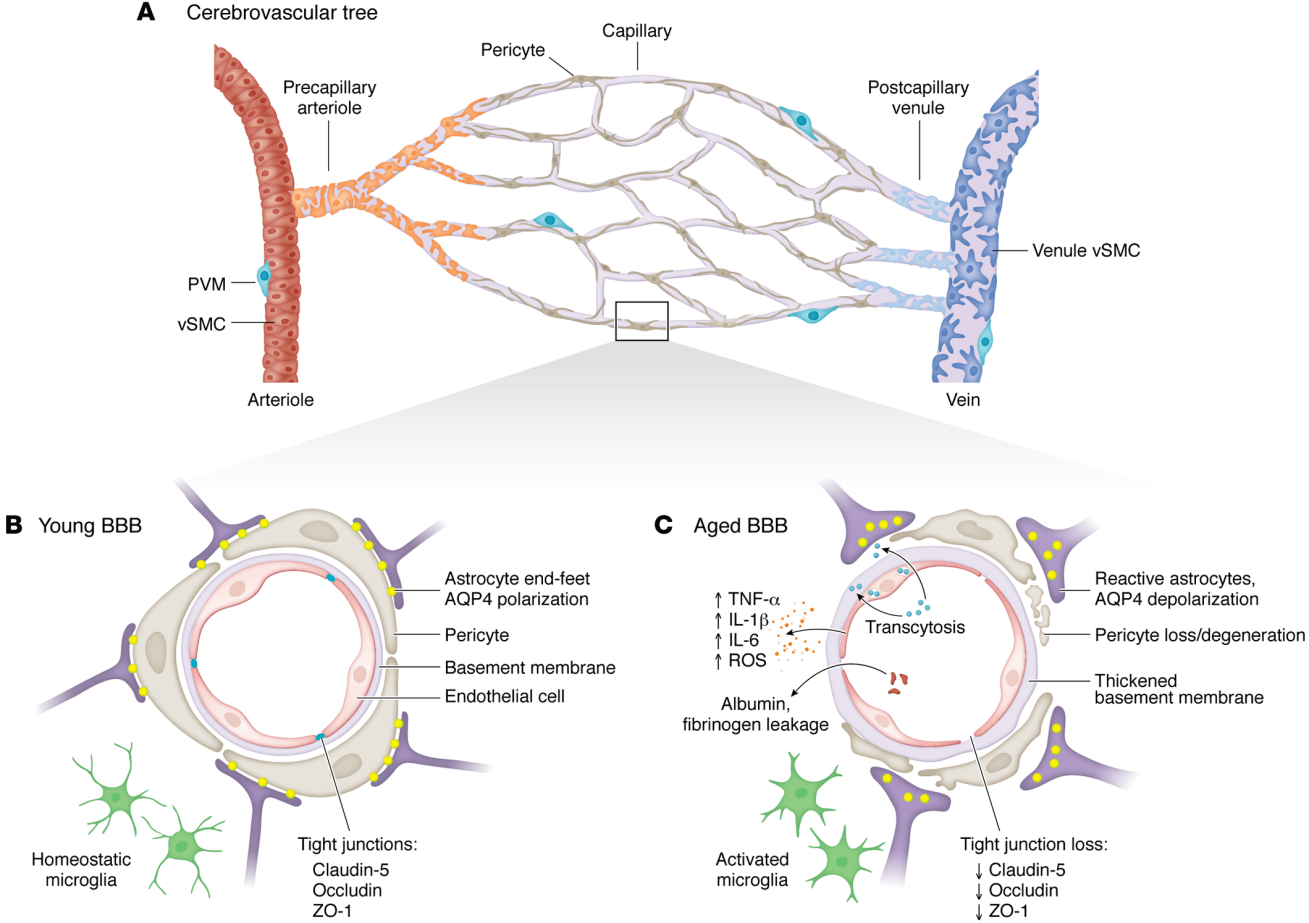
A schematic diagram of the young and aged BBB. (**A**) Schematic overview of the cerebral microvascular tree, including arterioles, precapillary arterioles, capillaries, postcapillary venules, and veins. Cellular composition varies along the vascular tree: vascular smooth muscle cells (vSMCs) are enriched in arterioles and are less so in veins, pericytes are abundant on capillaries, and perivascular macrophages (PVMs) localize to perivascular spaces. (**B**) In the young brain, the BBB consists of endothelial cells, pericytes, astrocyte end feet, and the basement membrane, which together maintain integrity. Endothelial tight junction proteins restrict paracellular permeability, while pericytes stabilize the vascular wall and astrocyte end feet exhibit polarized aquaporin-4 (AQP4) localization that supports BBB homeostasis. (**C**) In the aged brain, multiple alterations disrupt BBB function, including pericyte loss or degeneration, reduced expression of tight junction proteins, and increased transcytosis. Astrocytes become reactive and display AQP4 depolarization, and the basement membrane becomes thickened. These changes promote leakage of blood-derived proteins, such as albumin and fibrinogen, accompanied by microglial activation and increased inflammatory mediators, collectively contributing to neurovascular dysfunction.

The brain faces a unique dilemma: it has high energy demands but limited energy storage capacity. To function properly, it must receive a continuous supply of substrates for energy metabolism, primarily glucose. However, unlike many other cell types, neurons have a limited ability to draw on stored energy reserves and replenish them later (37). Therefore, adequate cerebral metabolism is essential for maintaining cerebral homeostasis. This balance is regulated by the neurovascular unit, a structural and functional entity composed of neurons, astrocytes, and vascular cells. The brain vasculature is organized as a highly specialized vascular network composed of arteries, arterioles, capillaries, venules, and veins, each with distinct structural and functional roles (Figure 1A). Large cerebral arteries and smaller arterioles regulate blood flow distribution and vascular resistance, thereby controlling cerebral perfusion. Arterioles are ensheathed by smooth muscle cells that mediate vasoconstriction and vasodilation in response to neuronal activity and metabolic demand. Capillaries, the brain microvasculature, are the primary sites of nutrient, metabolite, and gas exchange required for proper brain metabolism and constitute the core of the BBB (Figure 1B), a semipermeable interface that controls the exchange of substances between the blood and the brain while restricting the entry of toxins, pathogens, and immune agents (38). Downstream, postcapillary venules and venules facilitate immune cell trafficking, clearance of metabolic waste, and fluid drainage, while larger veins return deoxygenated blood to the systemic circulation (39).

Within the BBB, endothelial cells, which line the walls of brain capillaries, are specialized to create a highly selective and restrictive barrier that ensures proper brain function. The cells are flattened and form robust interendothelial tight junctions (38), which restrict paracellular diffusion and maintain BBB integrity through proteins such as claudin-5, occludin, JAMs, and the cytoskeletal scaffolding protein ZO-1 (40). Pericytes surround brain microvessels and help maintain BBB integrity, regulate blood flow, and coordinate endothelial–neural communication (41). These cells make close physical and functional contact with endothelial cells through peg-and-socket junctions and paracrine signaling (42). For example, endothelial cells secrete PDGF-BB, which binds to PDGFRβ on pericytes, driving their recruitment, survival, and function (43). Disruption of this signaling pathway results in vascular defects and related abnormalities (43–45). Astrocytes are also essential for BBB structure and function through their specialized end feet, which directly connect blood vessels with the neural environment. These end feet regulate water and ion homeostasis via channels such as AQP4 (46) and help maintain BBB integrity by releasing factors, such as sonic hedgehog, angiopoietin-1, and retinoic acid (47), that strengthen tight junctions and reduce vascular permeability.

Aging affects the cerebrovascular system at multiple levels, from large arteries to the microcirculation. Major intracranial arteries undergo structural stiffening and remodeling with age (48). Small penetrating arterioles are particularly vulnerable to chronic aging-related injury, exhibiting arteriolosclerosis, loss of smooth muscle cells, impaired autoregulation of cerebral blood flow, and reduced vasomotor responsiveness (49). The BBB also undergoes structural and functional alterations that compromise its integrity, including altered microvascular morphology/density, basement membrane thickening, endothelial cell senescence with disrupted tight junctions, pericyte loss, and increased BBB permeability (6, 7, 50, 51) (Figure 1C). Aging-associated BBB leakage arises from multiple interconnected mechanisms. Breakdown of tight junctions between endothelial cells reduces barrier selectivity, allowing unregulated paracellular diffusion of molecules (52). In parallel, age-related changes in BBB transport properties alter trafficking from selective receptor-mediated transcytosis toward nonspecific, caveolae-mediated transcytosis, further compromising BBB function (51). Concurrently, pericyte degeneration or loss promotes microvascular instability, capillary dilation, and increased BBB permeability (53, 54). Moreover, age-associated astrocyte changes, including end foot swelling, detachment and retraction from the vascular basement membrane, and loss of AQP4 enrichment at end foot membranes that anchor microvessels, further impair BBB integrity (55, 56). Another age-related cerebrovascular alteration is vascular calcification, which occurs mostly in small arteries/arterioles and capillaries, potentially reducing vascular compliance and impairing neurovascular coupling.

During the progression of AD, the BBB is continually compromised, exhibiting increased permeability, impaired transport mechanisms, and structural abnormalities (57). Dysfunction of all three major BBB cell types has been demonstrated in AD. For example, AD is characterized by loss of tight junction integrity and transporter dysfunction in endothelial cells, including reduced amyloid-β (Aβ) efflux and increased influx signaling, which together promote Aβ accumulation, BBB leakage, and vascular inflammation (58, 59). Pericyte degeneration also leads to increased BBB permeability, capillary dysfunction, and neurovascular instability. As pericytes are involved in LRP1-dependent Aβ clearance, pericyte dysfunction reduces Aβ peptide uptake (60, 61). Of note, *APOE4*, the strongest genetic risk factor for AD, is directly linked to cerebrovascular injury, including BBB leakage and pericyte dysfunction, beyond its effects on Aβ pathology (62, 63). Animal models expressing human *APOE4* exhibit cerebrovascular damage (64–66) and early BBB leakage (65).

Disruption of brain vasculature also contributes to the pathogenesis of cerebral amyloid angiopathy (CAA) and cerebral small vessel disease (CSVD), two age-related cerebrovascular disorders. In CAA, amyloid proteins build up in brain vessel walls. CAA and AD share Aβ pathology but the deposits differ: parenchymal plaques in AD are enriched in Aβ42, whereas vascular-wall deposits in CAA are enriched in Aβ40. In CAA, cerebrovascular walls and basement membranes show structural changes, and perivascular clearance of soluble Aβ is impaired (67–69). These clearance failures can create a feed-forward cycle: BBB leakage and inflammation damage small vessels, clearance worsens, and more vascular Aβ builds up. The vascular stress may also accelerate parenchymal Aβ42 deposition and interact with AD changes, reinforcing the frequent coexistence of CAA and AD. CSVD drives vascular dementia, which is the second most common cause of dementia after AD (70–72), implicated in approximately 70% of dementia cases (73). CSVD encompasses a group of pathological processes affecting small arteries, arterioles, and capillaries in the brain, leading to microvascular damage and structural changes such as BBB disruption, lacunar infarcts, microbleeds, and enlarged perivascular spaces (74, 75). These abnormalities strongly correlate with cognitive decline and often co-occur with AD pathology. Clinically, white matter hyperintensities (WMH) are widely used as imaging markers of CSVD. In large cohort studies, WMH were associated with elevated BBB leakage volume, highlighting a clear relationship between WMH severity and BBB breakdown (76, 77).

## Clinical observations supporting bone-cerebrovasculature interaction

Clinical and epidemiological evidence suggests a link between bone metabolism, cerebrovascular disease, and dementia. Multiple cross-sectional and longitudinal cohort studies have demonstrated an independent association between osteoporosis or low bone mineral density (BMD) and CSVD (78–82), the main contributor to vascular dementia. In a stroke cohort, lower BMD was associated with greater total CSVD burden and increased prevalence of most CSVD imaging markers (79). A more recent stroke-free cohort further demonstrated that osteoporosis was independently associated with increased CSVD burden, particularly in women with hip osteoporosis specifically linked to lacunes, severe WMH, and enlarged perivascular spaces (82). In addition, a longitudinal cohort study involving 3,651 older adults without dementia at baseline found that lower BMD was associated with an increased risk of incident dementia over a median follow-up of 11.1 years (83). Although the specific contribution of cerebrovascular pathology to the observed dementia risk remains unclear, these findings support a potential relationship between impaired bone metabolism, cerebrovascular dysfunction, and neurodegeneration. Additional clinical evidence for interactions between the skeleton and cerebrovascular system comes from osteoarthritis (OA) cohort studies. OA is a prevalent age-related joint degenerative disease. Although OA is primarily characterized by cartilage degeneration, accumulating evidence shows that subchondral bone alterations are a major driver of its pathogenesis (84–86). In a longitudinal cohort study, women with OA showed an increased risk of incident ischemic stroke compared with matched controls, with an adjusted hazard ratio of 1.34 during follow-up (87). Furthermore, a case-control study demonstrated that patients with knee OA had reduced cerebrovascular reactivity to hypercapnia, impaired cerebral autoregulation, and greater burden of WMH compared with healthy controls, suggesting early impairment of cerebrovascular function (88). These findings suggest that skeleton-related pathophysiological processes may contribute to cerebrovascular vulnerability. However, the causal role of subchondral bone alterations in cerebrovascular dysfunction has yet to be firmly established. In addition, several reported associations between osteoporosis or OA and cerebrovascular disease are based on cross-sectional data. Further well-designed longitudinal and mechanistic studies are needed to clarify causality and underlying pathways.

Consistent with the bone-cerebrovascular axis concept, clinical and preclinical studies suggest that antiosteoporosis therapy may influence cerebrovascular outcomes, though the evidence is more limited than that for systemic vascular effects. Bisphosphonates, potent antiresorptive agents commonly prescribed for osteoporosis, have been examined most extensively. Among patients with osteoporosis, long-term follow-up studies have suggested possible reductions in stroke and broader cardiovascular events in those prescribed bisphosphonates (89, 90). In a Hong Kong cohort of 121,492 older adults with osteoporosis or fragility fracture, nitrogen-containing bisphosphonate use was associated with a lower risk of dementia compared with both untreated patients and patients receiving nonbisphosphonate osteoporosis therapies (91). Importantly, the dementia outcome in this study included vascular dementia (91). In AD mouse models, alendronate, a commonly prescribed bisphosphonate, has consistently been shown to improve cognitive deficits and reduce Aβ accumulation and neuroinflammation (92, 93). However, direct evidence for its effects on BBB integrity, cerebral perfusion, or CSVD-related cerebrovascular dysfunction remains limited. Emerging evidence also supports cerebrovascular effects of teriparatide (synthetic PTH1–34), a potent anabolic agent. In rodent models of ischemic stroke, intermittent PTH1–34 treatment promoted angiogenesis and cerebral vessel formation, reduced infarct size, improved cerebral perfusion and local blood flow recovery, and attenuated BBB disruption and brain edema (94, 95). Furthermore, in a mouse model of AD, intermittent PTH1–34 administration reduced Aβ accumulation and neuroinflammation, while improving cognitive performance (96). Despite these promising preclinical findings, clinical evidence supporting cerebrovascular benefits of anabolic osteoporosis therapies remains limited. A notable clinical counterexample is odanacatib, a cathepsin K (CTSK) inhibitor. In a phase III clinical trial, odanacatib reduced vertebral, hip, and nonvertebral fracture risk but was associated with an increased stroke risk, leading to discontinuation of its development for osteoporosis (97, 98). This observation suggests that CTSK may play a context-dependent role in maintaining cerebrovascular homeostasis, consistent with evidence that circulating CTSK levels decline with age in humans (99).

## Experimental evidence that bone-derived factors influence cerebrovascular aging

### Age-related changes in bone.

The skeletal system is a dynamic structure that undergoes continuous remodeling (100), which is essential for maintaining skeletal health throughout life. Bone remodeling, which involves the coordinated activity of osteoclasts and osteoblasts (101), is initiated by the recruitment and activation of osteoclast precursors, which, under RANKL and M-CSF signaling, differentiate to TRAP^+^ mononuclear pre-osteoclasts (PreOCs) and subsequently fuse into mature multinucleated osteoclasts that resorb mineralized bone (102). This resorptive phase is tightly coupled with the recruitment and differentiation of osteoblast-lineage cells (103, 104), which form new bone at the same sites to preserve overall bone mass and quality.

With aging, bone remodeling becomes imbalanced and resorption exceeds formation, leading to reduced bone density, impaired structural integrity, and osteoporosis (101). The bone and bone marrow microenvironment also undergo substantial changes, including increased inflammatory cytokine production, oxidative stress, and cellular senescence (14, 15, 105). Cellular senescence represents a central pathogenic mechanism driving skeletal aging. Both human and animal studies have demonstrated that senescent cells of multiple types accumulate in bone and bone marrow with aging (14, 106). These senescent cells produce diverse bone-SASP factors (14, 15, 107, 108), which drive skeletal alterations through local paracrine effects. Another hallmark of skeletal aging is the expansion of marrow adipose tissue, which occupies over 70% of total marrow space by age 70 (109). Recent studies demonstrate that bone marrow adipocytes (BMAds) can develop a robust SASP, triggering secondary senescence in neighboring bone cells to promote bone loss (110). Interestingly, senescent BMAds secrete high levels of serum amyloid P (PTX2), which facilitates the aggregation of Aβ peptides into insoluble fibrils in the bone marrow. This skeletal amyloidosis disrupts bone remodeling and contributes to bone loss (13). Together with earlier studies showing amyloid precursor protein (APP) expression and pathological APP processing in bone cells (111–113), this work provides the first evidence that amyloid-related mechanisms contribute to bone-remodeling deterioration during aging and AD.

Age-related changes in the profile of bone- and bone marrow–derived signaling molecules contribute not only to skeletal deterioration but also to systemic dysfunction. Bone/bone marrow–derived factors that influence the cerebrovascular system are discussed below and summarized in Table 1.

### Osteoclast lineage–derived factors that influence cerebrovasculature.

In addition to their bone resorption function, osteoclasts secrete factors known as “clastokines” to maintain bone homeostasis (114). One of these clastokines is CTSK. The canonical role of CTSK is in bone resorption, specifically degrading the organic matrix (115). Clinical studies show that circulating CTSK levels decline with age (99). In addition, accumulating evidence indicates that CTSK contributes directly to arterial wall remodeling by degrading elastin and collagen, and CTSK-predigested elastin is substantially more prone to mineral deposition (116). While there is no direct demonstration that osteoclast-derived CTSK alone disrupts the cerebrovasculature, several human and experimental observations link CTSK levels to cerebrovascular risk. After experimental ischemia, *Ctsk*-knockout mice exhibit worse BBB disruption and hemorrhagic transformation (117), indicating CTSK deficiency may lead to BBB impairment. As noted above, a phase III trial of the selective CTSK inhibitor odanacatib as an antiosteoporosis drug was terminated after a signal of increased stroke, indicating that modulation of CTSK can intersect with cerebrovascular outcomes (97, 98). However, human expression datasets reveal detectable CTSK expression in brain regions, especially in the choroid plexus (118), suggesting that locally expressed CTSK may also influence cerebrovascular function (119). To definitively establish causality along the bone-cerebrovascular axis, osteoclast-specific conditional *Ctsk*-knockout models, combined with in vivo BBB functional assessments, will be required.

**Figure 2:**
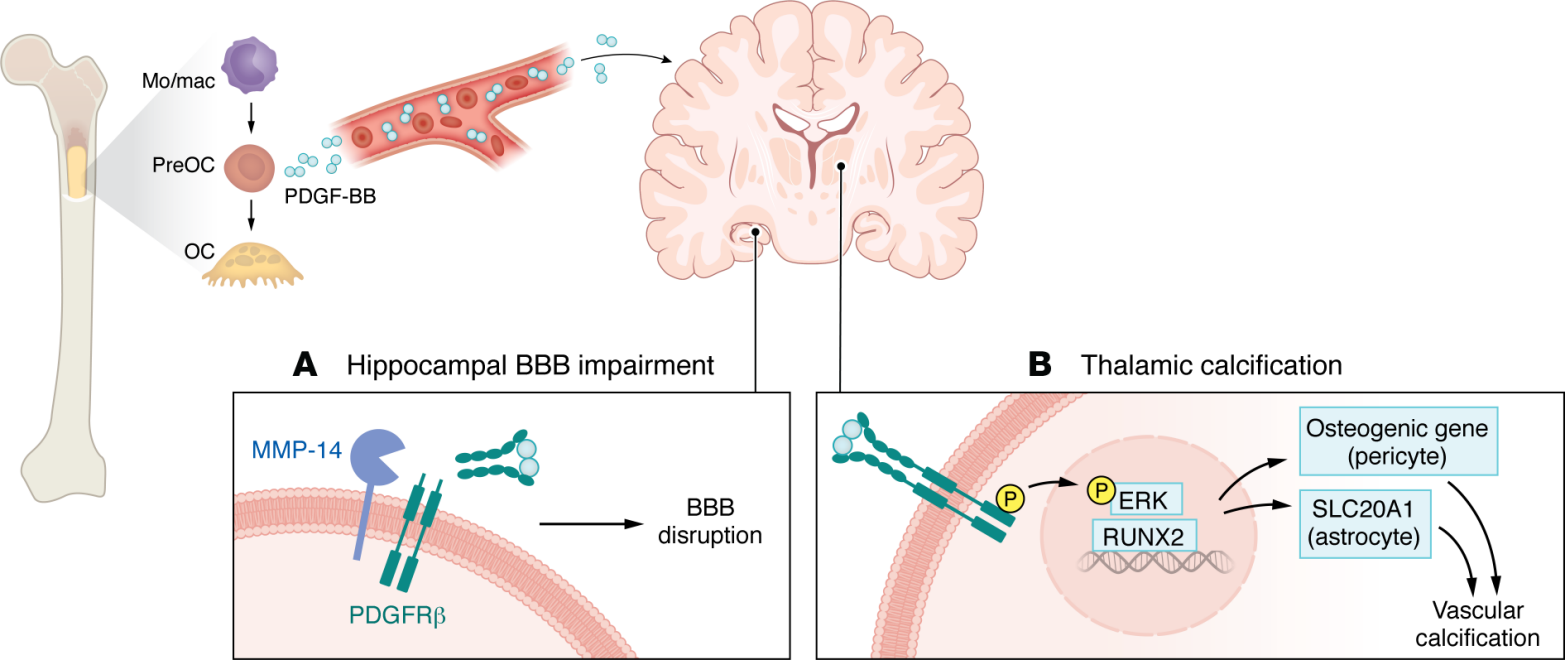
Bone-derived PDGF-BB drives region-specific cerebrovascular pathology. Skeletal PreOC-secreted PDGF-BB enters the circulation and acts on the brain vasculature. (**A**) Hippocampus: Sustained excessive PDGF-BB stimulation induces MMP-14 upregulation, promoting PDGFRβ shedding and weakening pericyte-endothelial interactions, leading to BBB damage. (**B**) Thalamus: Elevated PDGF-BB activates ERK/RUNX2 pathways and osteogenic gene programs in pericytes, along with astrocytic SLC20A1 upregulation, contributing to thalamic calcification.

During osteoclast differentiation, mononuclear PreOCs represent an intermediate stage with limited resorptive activity. An important factor secreted by PreOCs is PDGF-BB, which promotes angiogenesis and couples vascularization with osteogenesis during normal bone modeling and remodeling (120, 121). However, during aging, PreOC-derived PDGF-BB exerts detrimental effects on the skeleton. There is a marked increase in PDGF-BB secretion by PreOCs in aged mice and in those subjected to high-fat diet (5–7, 121), as well as in old humans (5) and patients with CSVD (122). We found that mice with transgenic overexpression of PDGF-BB in PreOCs (*Pdgfb^cTG^*) exhibited a 2~3-fold increase in serum PDGF-BB compared with wild-type littermates, recapitulating the aged phenotype. In contrast, aged mice with PreOC-specific knockout of PDGF-BB (*Pdgfb^cKO^*) showed normalized serum PDGF-BB levels, equivalent to those of young controls (6). These results support a bone-specific origin of elevated circulating PDGF-BB during aging. *Pdgfb^cTG^* mice developed spontaneous arterial stiffening, whereas *Pdgfb^cKO^* mice showed reduced age-associated arterial stiffening and preserved vascular compliance, indicating a role for PreOC-derived PDGF-BB in age-related arterial stiffening (5). Using these same mouse models, we further showed that PreOC-derived PDGF-BB is both sufficient and necessary to induce hippocampal BBB dysfunction, including reduced capillaries, pericyte loss, BBB leakage, and cognitive decline (6). Supporting our observation that elevated circulating PDGF-BB damages the BBB, a recent clinical study showed that higher circulating PDGF-BB levels correlate with WMH and small vessel damage in APOE4 carriers (122). Mechanistically, abnormally high levels of PDGF-BB can trigger MMP-14–mediated ectodomain shedding of PDGFRβ in pericytes, leading to pericyte degeneration and BBB dysfunction (6) (Figure 2A). Previous studies have shown that ADAM10 and ADAM17 can also mediate PDGFRβ ectodomain shedding (123, 124) under different pathological conditions. These distinct proteolytic mechanisms may generate soluble PDGFRβ (sPDGFRβ) fragments of varying size or composition. Importantly, clinical evidence indicates that cerebrospinal fluid (CSF) sPDGFRβ levels serve as a biomarker of pericyte degeneration and BBB disruption in aging individuals and patients with AD (125).

Elevated PDGF-BB also affects cerebral blood flow (CBF), as global CBF was markedly reduced in young *Pdgfb^cTG^* mice (126). The mice exhibited regional CBF deficits in the striatum and olfactory area, along with a higher oxygen-extraction fraction and increased metabolic rate of oxygen, indicative of a state of hypoperfusion and hypermetabolism. Elevated PDGF-BB may impair cerebral perfusion through multiple mechanisms, including pericyte loss and subsequent BBB dysfunction, as well as stiffening and calcification of small arteries and arterioles, which together could contribute to impaired cerebrovascular reactivity and dysregulated CBF.

Another defect associated with aberrantly elevated circulating PDGF-BB is cerebrovascular calcification (7), which is commonly detected in the elderly population under CT imaging (127) and at autopsy (128) and is associated with movement disorders, seizures, neuropsychiatric symptoms, and cognitive impairment. Calcification mainly occurs in the cerebral microvasculature and intracranial small arteries (7, 129, 130). Both aged WT male mice and *Pdgfb^cTG^* mice with elevated circulating PDGF-BB exhibited a higher incidence of cerebrovascular calcification compared with young mice, with lesions localized to the thalamic region (7). Mechanistically, elevated circulating PDGF-BB promotes osteogenic remodeling and mineral deposition in thalamic microvessels by activating PDGFRβ/ERK/RUNX2 signaling in cerebrovascular cells and enhancing astrocyte-mediated phosphate uptake and calcium phosphate deposition (7) (Figure 2B). The clinical relevance of cerebrovascular calcification is underscored by its association with primary familial brain calcification (PFBC), a rare neurodegenerative disorder characterized by bilateral calcification in the brain, particularly in the basal ganglia and thalamus (131). Genetic studies have identified mutations in genes such as *PDGFB*, *PDGFRB*, *SLC20A2*, and *XPR1* as major causes of PFBC (131). These genes are directly involved in PDGF-BB signaling and phosphate transport pathways (7, 131), supporting the hypothesis that dysregulation of PDGF-BB signaling plays a central role in age-related brain vascular calcification. The extent of calcification in intracranial arteries appears to correlate with atherosclerosis burden (132). Although PDGF-BB has been implicated in the development of atherosclerosis in extracranial vessels (5, 133), whether elevated bone-derived PDGF-BB exacerbates intracranial atherosclerosis remains unclear. In addition to cerebrovasculature, calcification of the choroid plexus is more frequently observed in clinical CT studies and has been linked to brain aging and neurological dysfunction (134, 135). Future studies should investigate whether PreOC-derived PDGF-BB contributes to choroid plexus calcification and associated blood-CSF barrier dysfunction.

### Osteoblast lineage–derived factors that influence cerebrovasculature.

The endocrine function of osteoblast-lineage cells, particularly osteoblasts and osteocytes, is well recognized (12, 35, 136). Particularly, OCN, which is specifically produced by osteoblasts, has been demonstrated to inhibit bone formation (12). OCN exists in distinct biochemical forms that differ in localization and biological activity. Fully carboxylated OCN has high affinity for hydroxyapatite and is predominantly retained in the bone matrix, where it contributes to mineral organization and bone material properties. In contrast, undercarboxylated OCN has lower mineral affinity and is released into the circulation (12). Loss- and gain-of-function studies demonstrate that this osteoblast-derived molecule, by binding to one of three receptors, regulates a broad array of systemic functions in rodents and primates (10, 137, 138), including glucose homeostasis (139, 140), energy expenditure (141), exercise capacity (142), blood pressure regulation (143), testosterone synthesis in the testis (144), muscle mass (145), and acute stress responses (146, 147). Furthermore, maternal- and embryo-derived OCN helps regulate systemic homeostasis in offspring during early life and adulthood (148).

Osteoblast-derived OCN, particularly in its undercarboxylated form, can cross the BBB and bind to G protein–coupled receptors GPR158 and GPR37 on neurons in regions such as the hippocampus, brainstem, and midbrain (149, 150). Both developmental and adult studies demonstrate that OCN is required for normal brain development, including hippocampal formation, and for the maintenance of cognitive function, with deficiency leading to impaired spatial learning and memory (136). Notably, clinical studies indicate that circulating OCN declines with age and AD in both men and women (151) and correlates positively with cognitive performance (142, 152). In accord with those results, experimental restoration of OCN reverses age-related cognitive decline in animal models (142, 153, 154). Moreover, in patients with acute ischemic stroke, higher serum OCN levels during the acute phase have been associated with greater functional improvement (155). Although direct experimental evidence supporting the role of OCN in regulating BBB integrity and cerebral microvascular function remains limited, its ability to cross the BBB, modulate neuroinflammation and neurotransmission, and improve vascular function more broadly suggests that it may directly or indirectly influence BBB function and brain aging. Future studies are needed to define whether bone-derived OCN exerts a direct effect on the cerebrovasculature or acts indirectly through neuronal, metabolic, or systemic vascular pathways.

FGF23 is another hormone predominantly produced by osteocytes and osteoblasts (156). Its canonical endocrine function is to regulate phosphate and vitamin D metabolism (156). During aging, circulating FGF23 levels tend to increase in both men and women (157). Numerous clinical studies have identified elevated circulating FGF23 levels as a potential biomarker in patients with cognitive and cerebrovascular impairment (158–160), and it has been associated with stroke risk (161, 162), subclinical cerebrovascular damage (163), and incident dementia (159). Animal studies further show that impaired FGF23 signaling can lead to neuronal dysfunction and behavioral abnormalities (164, 165), but whether bone-derived FGF23 regulates cerebral microvascular function is unknown.

DKK1, predominantly produced by osteoblast-lineage cells (166), suppresses canonical Wnt signaling by targeting the LRP5/6 coreceptor complex. This pathway is critical for cerebrovascular homeostasis, as Wnt/β-catenin signaling in brain endothelial cells supports BBB development and maintenance (167). Clinical studies show that circulating DKK1 levels are elevated in older adults compared with younger individuals and in patients with both acute and chronic cerebrovascular disease (168–170). In a CSVD model induced by multifocal cerebral microinfarctions, circulating DKK1 increased and remained elevated before gradually returning toward baseline (171). Conditional DKK1 induction further exacerbates vascular permeability and impairs acute cerebrovascular reactivity and brain perfusion (172). Importantly, bone marrow–restricted DKK1 induction is sufficient to accelerate subacute injury progression, providing evidence that bone/bone marrow–derived DKK1 directly contributes to cerebrovascular dysfunction (172).

Sclerostin, primarily secreted by osteocytes, is another bone-derived Wnt antagonist (173). Serum sclerostin levels increase markedly across the adult lifespan, with studies reporting an approximately 2.4-fold increase in women and 4.6-fold increase in men between 21 and 97 years of age (174). Recent work has demonstrated that bone-derived sclerostin can cross the BBB and is implicated in the pathophysiology of various brain conditions, including brain shrinkage and vascular calcification (175). Importantly, increased sclerostin expression was observed only in bone but not in brain or other tested peripheral tissues during aging, supporting bone as the primary source of elevated sclerostin detected in the brain (176). Gain- and loss-of-function studies further show that elevated osteocyte-derived sclerostin impairs synaptic plasticity and memory, whereas osteocyte-specific deletion, bone-targeted knockdown, or treatment with sclerostin-neutralizing antibody improves cognitive outcomes (176). Importantly, neuroimaging studies have shown that elevated circulating sclerostin levels are associated with white matter damage (177), cognitive decline (178), and neurodegenerative diseases (179). It will be interesting to determine whether elevated sclerostin directly influences BBB function.

LCN2, also known as oncogene 24p3 or neutrophil gelatinase-associated lipocalin, is another secreted factor produced by osteoblasts, with systemic effects. In mice, osteoblast-specific deletion of *Lcn2* reduces circulating LCN2 levels by approximately 67% (180), indicating that osteoblasts are a major source of circulating LCN2. During aging and dementia, serum LCN2 levels increase in healthy men and women (181, 182). Circulating LCN2 can cross the BBB and act on multiple brain regions (180, 183). Clinical studies using imaging techniques have shown that LCN2 is closely associated with the progression of various neurodegenerative and neurological disorders, including AD (184), ischemic stroke (185), and CSVD-associated white matter damage (186). Experimental studies further demonstrate that elevated circulating LCN2 exacerbates neurovascular injury responses and impairs BBB integrity by disrupting tight junctions and inflammatory signaling pathways (187, 188). Recent work in thrombolysis-treated ischemic stroke implicates LCN2 in endothelial ferroptosis–mediated BBB disruption (188). These findings suggest that circulating LCN2 directly affects the neurovascular interface. However, it remains unclear whether osteoblast-derived LCN2 specifically contributes to cerebrovascular dysfunction, as LCN2 is also locally expressed in brain cells under inflammatory or ischemic conditions (185). Nonetheless, because circulating LCN2 can cross the BBB, it is important to determine whether osteoblast-specific LCN2 mediates its detrimental cerebrovascular effect using osteoblast-specific LCN2 gain- and loss-of-function approaches.

In addition to the factors described above, other bone- and marrow-associated signals may contribute to neurovascular aging. For example, TGF-β, which is stored in bone matrix and released during osteoclastic bone resorption (189), exerts broad vascular effects by regulating multiple cerebrovascular cells (190). Similarly, BMPs promote osteogenic remodeling of vascular cells and contribute to vascular calcification (191). Moreover, skeletal aging is associated with the accumulation of senescent bone and marrow cells that produce diverse inflammatory cytokines or bone-SASP factors (14), which may amplify systemic inflammation and promote cerebrovascular dysfunction (192). Age-related remodeling of the hematopoietic niche may further influence the brain through altered immune cell output and inflammatory cytokine production (193). However, as these molecules are widely produced across multiple tissues, including the brain, the extent to which bone-specific signals uniquely contribute to the bone-cerebrovascular axis remains to be determined. In addition to proteins, exosomes derived from bone marrow stem cells (BMSC-exos) are emerging as key mediators in the bone-brain axis due to their ability to traverse the BBB and deliver bioactive cargoes, including miRNAs, proteins, and lipids (194). Multiple preclinical studies have shown that BMSC-exos protect endothelial cells, promote cerebrovascular angiogenesis, and stabilize the BBB, thereby exerting a beneficial effect on cerebrovascular function (194, 195). Notably, these studies have largely relied on the exogenous administration of BMSC-exos in animal models; whether endogenous vesicles derived from bone marrow stem cells contribute to the bone-cerebrovascular axis during aging or disease conditions remains unclear.

While here we primarily emphasize long-distance, circulating signals derived from bone and bone marrow, emerging evidence suggests that skull-associated bone and bone marrow may provide a more localized route for bone-cerebrovascular interactions through anatomically restricted pathways. The skull bone marrow is now recognized as a specialized niche that modulates CNS immune homeostasis via skull-meningeal vascular channels, which enable the direct trafficking of immune cells between the calvarial marrow and the dura mater (196–200). Skull marrow may also communicate with the CNS through local, CSF-associated signaling, potentially allowing soluble factors to access meningeal and perivascular space (197, 201, 202). These findings support a model in which the calvarial bone marrow engages in both cellular and humoral crosstalk with adjacent neurovascular compartments, complementing systemic bone-derived signaling pathways.

## Sex differences in the bone-cerebrovascular axis

Sex differences influence both the skeleton and the cerebrovasculature during aging. In women, the decline in estrogen at menopause leads to a marked increase in bone resorption relative to formation, resulting in accelerated bone loss and high-turnover osteoporosis. In men, the gradual age-related decline in androgens produces a slower and more modest increase in bone turnover, leading to a later and less abrupt decline in bone mass (203). Similarly, the age-related decline in estrogen in women is associated with endothelial dysfunction, reduced cerebrovascular reactivity, increased BBB permeability, and impaired neurovascular coupling (204). Consequently, the incidence of stroke rises sharply in postmenopausal women, eventually exceeding that of men, particularly at older ages (205). Women also show a higher prevalence of CSVD and white matter injury in later life, which are key contributors to vascular cognitive impairment and dementia (206).

As estrogen deficiency after menopause accelerates bone remodeling, sex differences in bone-derived factors likely become more pronounced with aging. Estrogen suppresses bone turnover and OCN release (207), suggesting that OCN-mediated regulation of brain function may diverge between sexes during aging. Circulating sclerostin levels are generally higher in men than in women; however, in women sclerostin levels increase markedly after menopause, likely reflecting the loss of estrogen-mediated inhibition of *SOST* (encoding sclerostin) (174). In addition, postmenopausal females exhibit a marked rise in senescence-associated bone turnover and inflammatory secretome (208, 209), which may influence systemic inflammation and vascular aging, including cerebrovascular dysfunction. A particularly striking sex difference along the bone-cerebrovascular axis involves PDGF-BB and brain vascular calcification: we observed brain calcified nodules in 80% of aged male mice but in none of the age-matched female mice (7). This aligns with a large human cohort study showing that most forms of intracranial calcification, including vascular calcification, occur in aged men (134). The findings suggest that sex-specific, age-dependent alterations in PDGF-BB may contribute to differential susceptibility to brain vascular calcification. More broadly, they underscore the importance of incorporating sex as a biological variable in studies of the bone-cerebrovascular axis. A better understanding of sex differences in bone-derived signals may inform personalized strategies to preserve skeletal integrity and cerebrovascular health during aging.

## Conclusion and perspective

The skeleton has emerged as a previously underappreciated regulator of cerebrovascular function. With aging-associated changes in bone remodeling, bone and bone marrow cells undergo corresponding shifts in their secretome, which influence cerebrovascular integrity and contribute to the bone-cerebrovascular axis. Despite recent advances discussed in this Review, the field remains in its infancy, and many fundamental questions remain unresolved. A key question concerns the identity of the primary cellular targets underlying BBB dysfunction. One plausible possibility is that bone-derived factors act directly on endothelial cells via specific receptors, thereby altering their structure and function, for example, by reducing the tight junction integrity and inducing cellular senescence. Alternatively, these factors may traverse the endothelial barrier and act on pericytes and other perivascular niche cells, as aging alters protein transcytosis mechanisms in ways that allow certain circulating proteins to cross the endothelial layer of the BBB more readily. Our finding that sustained PDGF-BB elevation directly targets pericytes to induce PDGFRβ cleavage supports this latter possibility.

It is also important to note that, although this Review emphasizes increased BBB permeability that allows detrimental circulating factors to access the brain, aging may simultaneously reduce the availability and regulated transport of beneficial nutrients, hormones, and trophic factors across the cerebrovascular interface (50, 51, 210). Therefore, aging-related cerebrovascular dysfunction may result not only from increased exposure to detrimental circulating pro-aging factors but also from reduced delivery of beneficial plasma-derived nutrients, trophic signals, and antiaging factors. Consistent with this concept, exposure to young blood or plasma improves synaptic plasticity and cognitive function in aged mice (8). Importantly, age-related blood–cerebrovascular communication is bidirectional in its consequences. On one hand, circulating factors influence vascular and neural function within the CNS; on the other hand, cerebrovascular dysfunction can alter systemic homeostasis by disrupting neuroendocrine signaling, autonomic regulation, and immune crosstalk. These reciprocal interactions may amplify pathological processes across organ systems, reinforcing the concept of a systemic aging network. Although here we focus primarily on BBB dysfunction, other circulation-CNS interfaces should also be considered, such as the blood-CSF barrier at the choroid plexus. The choroid plexus regulates CSF production, solute transport, waste clearance, and immune surveillance, and its structure and permeability change with aging and AD. Future studies should determine whether skeletal aging and bone-derived signals influence the blood-CSF barrier, choroid plexus calcification, and CSF-mediated neuroimmune homeostasis.

From a translational perspective, several avenues warrant exploration. First, bone- and bone marrow–derived factors detectable in blood could serve as minimally invasive biomarkers for early neurovascular dysfunction and prodromal neurodegenerative disease. Establishing temporal and causal relationships between skeletal signaling changes and cognitive decline will require longitudinal studies and refined animal models. Second, targeting skeletal endocrine function, through pharmacologic, lifestyle, or niche-specific interventions, could offer new strategies to preserve neurovascular health, but such approaches must account for systemic effects and tissue specificity. Importantly, neurovascular aging extends beyond cognition to include impairments in motor and autonomic function, broadening the scope of the bone-cerebrovascular axis to encompass multiple neural systems.

## Conflict of interest

The authors have declared that no conflict of interest exists.

## Funding support

This work is the result of NIH funding, in whole or in part, and is subject to the NIH Public Access Policy. Through acceptance of this federal funding, the NIH has been given a right to make the work publicly available in PubMed Central.

- National Institutes of Health grants R01AG068226 and R01AG072090 to MW.

## Figures and Tables

**Table 1:**
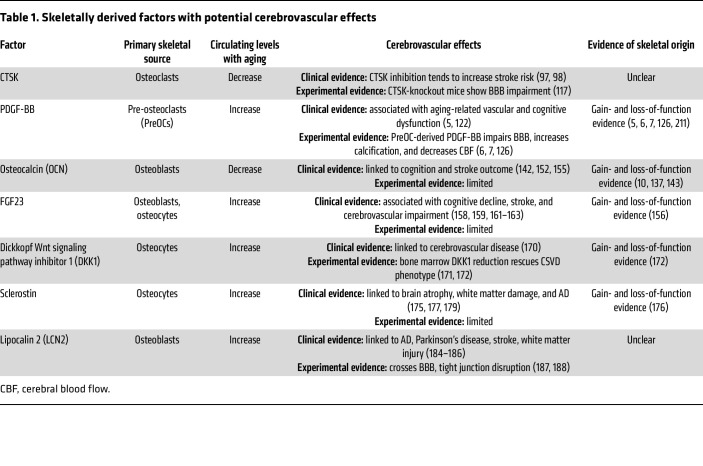
Skeletally derived factors with potential cerebrovascular effects

## Version 1 07/15/2026

Electronic publication
